# Negative Pleural Fluid Cultures among Patients with Pleural Effusion in a Tertiary Care Hospital: A Descriptive Cross-sectional Study

**DOI:** 10.31729/jnma.6758

**Published:** 2022-05-31

**Authors:** Srijana Ranjit, Amit Kumar Singh, Ishu Shrestha, Anu Radha Twayana, Prabha Bhandari, Shisir Siwakoti, Shrijana Singh

**Affiliations:** 1 Department of Microbiology, Dhulikhel Hospital, Kathmandu University, Dhulikhel, Kavrepalanchok, Nepal; 2 Department of General Surgery, Dhulikhel Hospital, Kathmandu University, Dhulikhel, Kavrepalanchok, Nepal; 3 Kathmandu University School of Medical Sciences, Dhulikhel, Kavrepalanchok, Nepal

**Keywords:** *empyema*, *microbiology*, *pleural effusion*, *thoracocentesis*

## Abstract

**Introduction:**

A systematic approach to analysis of the fluid in conjunction with the clinical presentation allows clinicians to diagnose the cause of an effusion, narrow the differential diagnoses, and design a management plan. However, the number of cases where pleural fluid examination gives no proper diagnosis is depressingly high. This study aims to find out the prevalence of negative pleural fluid cultures among patients with pleural effusion in a tertiary care hospital.

**Methods:**

This was a descriptive cross-sectional conducted among 273 patients with pleural effusion admitted to a tertiary care hospital between January, 2019 and February, 2020. Ethical clearance was taken from the Institutional Review Committee (Reference number: 134/20). Convenience sampling was done. All patients whose pleural fluid was sent for analysis during the study period were included in the study. Pleural fluid analysis was done, and data were analysed using Statistical Package for the Social Sciences 25.0. Point estimate was done at a 95% Confidence Interval along with frequency and percentages for binary data.

**Results:**

Among 273 pleural fluid cultures from patients with pleural effusion, negative pleural fluid cultures were seen in 269 (98.53%) (97.12-99.96 at a 95% Confidence Interval).

**Conclusions:**

Our study reported that the prevalence of negative pleural fluid cultures was higher when compared to similar studies conducted in similar settings. The routine pleural fluid analysis could add a very little to the diagnosis and management of pleural effusion.

## INTRODUCTION

The pleural space is generally sterile, but once pleural fluid has accumulated, it is easily infected. Pleural effusions develop in up to 44%-60% of patients with community-acquired pneumonia (CAP).^1,2^ Negative pleural fluid culture ranges from 90%-98% among patients with pleural effusion.^2,3^ The utility of pleural effusion study as a tool to diagnose the disease condition or help in management of pleural effusion is doubtful.^3,4^

To our knowledge there is no study done in Nepal on the impact of pleural effusion bacteriology study, its prevalence and its supportive use on the treatment plans. The number of cases where pleural fluid examination yields no diagnosis is depressingly high.^5^

This study aims to find out the prevalence of negative pleural fluid cultures among patients with pleural effusion in a tertiary care hospital.

## METHODS

This descriptive cross-sectional study was conducted between January, 2019 and February, 2020 in Dhulikhel Hospital. Ethical clearance was obtained from Institutional Review Board (Reference number: 134/20). All patients presenting to the hospital during the study period as a diagnosed case of pleural effusion or were diagnosed to have pleural effusion with the help of clinical, laboratory or radiological investigation who satisfied the inclusion and exclusion criteria were taken in study. All the patients with pleural effusion having complete data were included. Data of intubated patients and surgical patients (hemodynamically unstable), patients who had already undergone pleural tapping in past and already on treatment were also excluded. Convenience sampling was done. The sample size was calculated using the formula:

n = (Z^2^ × p × q) / e^2^

  = (1.96^2^ × 0.5 × 0.5) / 0.1^2^

  = 97

Where,

n = minimum required sample size
Z = 1.96 at 95% Confidence Interval (CI)
p = prevalence taken as 50% for maximum sample size calculation
q = 1-p
e = margin of error, 1%

Since the convenience sampling was used in the study, doubling the sample size, we got 194. However, 273 cases were taken into the study. Patient of both sexes who were diagnosed to have pleural effusion with clinical and radiological evaluation and ultimately confirmed by pleurocentesis. Sputum and pleural fluid analysis were done as a part of treatment after getting written informed consent. All the participants were studied in detail with clinical history, physical examination, and laboratory investigations.

Data were collected through hospital records, and management details were obtained. Data was retrieved in the paper-based questionnaire. The data were entered and analysed using the Microsoft Excel and the Statistical Package for the Social Sciences (SPSS) version 25.0. Point estimate was done at a 95% Confidence Interval along with frequency and percentages for binary data.

## RESULTS

Among 273 pleural fluid cultures from patients with pleural effusion, negative pleural fluid cultures was seen in 269 (98.53%) (97.12-99.96 at a 95% Confidence Interval). One hundred sixty-nine (62.82%) patients received antibiotics and 72 (26.76%) patients received Anti-tubercular Therapy (ATT) for their treatment regardless of the diagnostic output from the microbiological examinations. Negative pleural fluid culture was more common among males seen in 182 (67.65%) and the M:F ratio was 2.09:1. The mean age of the patients was 49.29±22.71 years (Table 1).

**Table 1:** Socio-demographic details (n = 269).

Variables		n (%)
**Gender**	Male	182 (67.65)
	Female	87 (32.35)
**Age (years)**	<20	35 (13.01)
	20-40	61 (22.67)
	40-60	76 (28.26)
	>60	97 (36.06)

Among all the patients with negative pleural effusion culture, 2 (0.74%) had a family history of tuberculosis and 83 (30.85%) patients had a past history of tuberculosis (Table 2).

**Table 2:** Past medical history of patients (n = 269).

Medical history	n (%)
Past history of tuberculosis	83 (30.85)
Family history of tuberculosis	2 (0.74)

Pleural effusion in our study sample with negative culture was more common on the right side 137 (50.93%) followed by the left side 76 (28.26%) and bilateral 56 (20.81%) respectively. In gram stain, 7 (2.61%) samples were gram-positive and 262 (97.39%) were gram-negativ. None of the samples showed acid-fast positivity (Table 3).

**Table 3:** Clinical and bacteriological characteristics of patients (n = 269).

Variables		n (%)
**Site**	Left	76 (28.26)
	Right	137 (50.93)
	Bilateral	56 (20.81)
**Gram stain**	Positive	7 (2.61)
	Negative	262 (97.39)
**AFB stain**	Positive	-
	Negative	269 (100)
**Clinical diagnosis of tubercular effusion**		83 (30.85)

Among all 269 patients, 83 (30.85%) patients were diagnosed to have tuberculosis clinically irrespective of pleural effusion bacteriology analysis and out of this, 72 patients (86.74%) needed antitubercular drugs (Table 4).

**Table 4:** Conservative management (n = 269).

Variables	n (%)
Clinical diagnosis of tubercular effusion	83 (30.85)
Patients managed with anti-tubercular therapy (ATT)	72 (86.74)
Patients requiring antibiotics (Other than ATT)	169 (62.82)

## DISCUSSION

We found that the prevalence of the negative pleural fluid bacterial cultures from pleural effusion is very high as 269 (98.53%) out of 273 patients had negative pleural fluid bacteriological cultures. One hundred sixty nine (61.90%) patients out of 273 patients received antibiotics and 72 (86.74%) patients received ATT for their treatment, which could highlight the possible limited value of pleural fluid bacteriology study as a diagnostic tool in the initial management of pleural effusion as regardless of the diagnostic output from the microbiological examinations. The low yield for diagnosis, less efficient rapid diagnosis (Gram stain), inability to assist in making therapeutic decisions, low culture-positive rate, minimal additional information by the study and no impact on the outcome of the disease limits the effectiveness of this test. Similar findings were seen in 1637 samples of pleural fluid cultures where only 14 patients (1.1%) had positive bacterial culture.^2^

One of the largest systemic reviews published in 2019 which included 10241 patients with pleural effusion showed that in more than two-fifth of cases the pleural fluid cultures showed no growth and the antimicrobial treatment was totally empirical.^6^ In our study, we found that the culture positivity was only 1.46%. The lower yield of culture positivity was also supported by a study done in 2006 on 259 pleural fluid samples^5^ and another study done in 85 consecutive patients with a chest infection and pleural effusion^7^ who found 19.3% and 25% positive microbiological results, respectively. Their study shows a higher rate of positive culture reports than ours because patients in their series were sicker and the majority had empyema and loculations in the lungs. The culture positivity rate varies in different studies and factors contributing to this could be the severity of the patient taken in to study and also the practice of antibiotic administration.

In our study, Gram stain and Acid Fast Bacilli stain showed low diagnostic yield. This was also seen in many other series of studies done in past.^5,6,8,9^ This showed that males 182 (67.65%) were more commonly affected than females 87 (32.35%) with a male:female ratio of 2.09:1. This result is comparable to the studies done in a tertiary care hospital in India^10^ which showed male:female ratio of 1.47:1. Lower rate in females could be due to the lower health attention-seeking behaviour and lower admission rate in females in developing country like ours.

The limitation of the study is that it was a retrospective single hospital-based study with convenience sampling, and this could limit the applicability of the results to the larger group of population. Nevertheless, the comparable findings of the study with other studies is a remarkable strength.

## CONCLUSIONS

Our study reported that the prevalence of negative pleural fluid cultures was higher when compared to similar studies conducted in similar settings. This could highlight that the routine pleural fluid bacteriology adds very little to the management of pleural effusion as there is very high negative pleural fluid culture and regardless of the bacteriological profile more than half of the patients received treatment with antibiotics. The culture of pleural fluid doesn't dictate the use or changes in antibiotics nor gives any additional information. Based on our study we would like to recommend that pleural fluid studies shouldn't be ordered routinely unless the clinicians suspect an empyema. Further studies on role of pleural fluid cultures in management is warranted.
